# Antiphospholipid syndrome-associated myocarditis: role of ^18^F-FDG PET/CT in differentiation from immune-mediated or viral etiologies

**DOI:** 10.1097/MS9.0000000000005212

**Published:** 2026-06-03

**Authors:** Naveed Ahmad, Muhammad Qaseem, Imad Khan, Shehbaz Ali, Abdul Haseeb Hasan

**Affiliations:** a Department of Medicine, Khyber Medical College, Peshawar, Pakistan; b Department of Medicine, Northwest School of Medicine, Peshawar, Pakistan; c Department of Research, Oli Health Magazine Organization, Kigali, Rwanda; d Department of Internal Medicine, Sentara Northern Virginia Medical Center, Woodbridge, VA, USA; e Department of Medicine, King Edward Medical University, Lahore, Pakistan

*To the Editor,*

Antiphospholipid syndrome (APS)-associated myocarditis is a rare inflammatory cardiac manifestation of APS, a prevalent autoimmunity-related thrombo-inflammatory disorder characterized by chronic antiphospholipid antibody production, leading to clotting and pregnancy-related complications. This disease process is primarily mediated by antibodies targeting endothelial cells through complement pathways, directly damaging cardiomyocytes and leading to focal necrotic inflammation without thrombosis. Clinically, it is usually associated with high troponin values, regional wall motion abnormalities, and preservation of the ejection fraction, mimicking the myocarditis seen with immune checkpoint inhibitors and viral infections. It is estimated that it has a prevalence of 50 out of every 100 000 people and even higher mortality rates, thus contributing considerably to silent cardiac diseases. A recent case series reported in early 2026 highlights the lack of awareness of this disease and the underdiagnosis of the autoimmune etiology of myocarditis. To diagnose this condition against immune-mediated or viral causes, the diagnostic test of choice is ^18^F-FDG PET/CT, while cardiac MRI and endomyocardial biopsy remain crucial alternatives[1].

^18^F-FDG PET/CT is important for identifying myocardial inflammation because it detects areas of increased glucose uptake that correspond to areas of active inflammation. Therefore, it is a valuable tool for distinguishing between myocarditis associated with APS and myocarditis arising from viral infections or immunity issues. The efficacy of this diagnosis lies in the use of the two parameters, perfusion and metabolism, which allow for the detection of worrying signs of inflammation, despite the patient having a normal ejection fraction or experiencing non-specific symptoms. Appropriate nutritional guidelines ensure good sensitivity in excluding the physiological uptake of glucose in heart tissue. Importantly, ^18^F-FDG PET/CT results provide information that guides the immediate implementation of immunosuppressive strategies. Interestingly, in patients with inflammatory cardiomyopathy, the combination of a perfusion defect and increased FDG uptake had a hazard ratio of 3.9 for an unfavorable outcome[2].

Recent studies have proven the efficacy of ^18^F-FDG PET/CT in differentiating between inflammatory and structural heart diseases using metabolic and anatomical approaches. Studies related to PET/MR imaging show higher metabolism in cases of malignancy or inflammation than in benign lesions. Moreover, better patient preparation techniques make images clearer and reduce physiological myocardial uptake, which is important for a proper diagnosis. One study demonstrated a mean SUVmax of 13.2 ± 6.2 in malignant cases versus 2.0 ± 0.9 in benign cases, with a diagnostic sensitivity of 100% and specificity of 92% using an SUVmax threshold of ≥5.2[3]. Furthermore, one study showed that a dietary regimen reduced myocardial uptake in more than 80% of the patients[4].

Despite the rising trend, however, the diagnosis of inflammatory heart disease using ^18^F-FDG PET/CT combined with computed tomography angiography (CTA) faces several problems. First, variations in patient preparation, the imaging procedure itself, and diagnostic criteria might affect the consistency of the results obtained from various healthcare providers. In addition, normal heart tissue FDG accumulation and errors, such as misregistration or poor FDG suppression, may lead to false-positive or inconclusive results. The utilization of contrast-enhanced CTA can be limited in patients with kidney problems or a history of allergies. It is significant that semi-quantitative evaluation methods have not been universally validated, as one study has shown that SUVs greater than 3.3 can help avoid false positives in infective endocarditis[5].

Myocarditis associated with APS is a disease entity that does not receive widespread recognition and is difficult to diagnose. The application of ^18^F-FDG PET/CT offers valuable insights, allowing for the detection of myocardial inflammation and differentiation from other etiologies. Early adoption of this method will enable timely immunosuppressive interventions and improved outcomes. Standardization and recognition are critical factors for optimal applications.

## Data Availability

Data sharing is not applicable to this article, as no datasets were generated or analyzed during the current study.
